# Scatterometry-Based Monitoring of Laser-Induced Periodic Surface Structures on Stainless Steel

**DOI:** 10.3390/s25165031

**Published:** 2025-08-13

**Authors:** Agustín Götte, Marcelo Sallese, Fabian Ränke, Bogdan Voisiat, Andrés Fabián Lasagni, Marcos Soldera

**Affiliations:** 1 Institut für Fertigungstechnik, Technische Universität Dresden, George-Bähr-Str. 3c, 01069 Dresden, Germany; 2 Fraunhofer-Institut für Werkstoff- und Strahltechnik IWS, Winterbergstr. 28, 01277 Dresden, Germany

**Keywords:** scatterometry, laser-induced periodic surface structures (LIPSS), ultrashort pulsed laser processing, in-line process monitoring

## Abstract

Monitoring of laser-based processes is essential for ensuring the quality of produced surface structures and for maintaining the process stability and reproducibility. Optical methods based on scatterometry are attractive for industrial monitoring as they are fast, non-contact, non-destructive, and can resolve features down to the sub-microscale. Here, Laser-Induced Periodic Surface Structures (LIPSS) are produced on stainless steel using ultrashort laser pulses in combination with a polygon scanning system. After the process, the fabricated LIPSS features are characterized by microscopy methods and with an optical setup based on scatterometry. Images of the diffraction patterns are collected and the intensity distribution analyzed and compared to the microscopy results in order to estimate the LIPSS height, spatial period, and regularity. The resulting analysis allows us to study LIPSS formation development, even when its characteristic diffraction pattern gradually changes from a double-sickle shape to a diffuse cloud. The scatterometry setup could be used to infer LIPSS height up to 420 nm, with an estimated average error of 7.7% for the highest structures and 11.4% in the whole working range. Periods estimation presents an average error of ~5% in the range where LIPSS are well-defined. In addition, the opening angle of the LIPSS was monitored and compared with regularity measurements, indicating that angles exceeding a certain threshold correspond to surfaces where sub-structures dominate over LIPSS.

## 1. Introduction

Laser-induced periodic surface structures (LIPSS) are self-organized nano- and microscale patterns that emerge on a wide range of materials when irradiated with intense laser pulses [1,2]. Owing to their versatility in tailoring surface properties, LIPSS have attracted considerable attention for applications in optics, electronics, fluidics, tribology, and biomaterials [3,4,5,6,7,8]. The formation of LIPSS is influenced by multiple parameters, including the substrate material, laser fluence, number of applied pulses, pulse duration, wavelength, and polarization [9,10]. By precisely controlling these factors, the morphology of the resulting structures can be tuned to achieve desired surface functionalities. LIPSS typically appear as quasi-periodic, grating-like topographic features on the material surface and they are generally classified based on their spatial period (*Λ*) and orientation relative to the laser polarization. The most commonly observed types are low spatial frequency LIPSS (LSFL) and high spatial frequency LIPSS (HSFL) [11]. LSFL, with spatial periods larger than half the laser wavelength, can be oriented perpendicular (LSFL-I) or parallel (LSFL-II) to the laser polarization, depending on the specific formation mechanism. In contrast, HSFL exhibit spatial periods smaller than half of the laser wavelength and are typically categorized as deep or shallow, based on whether their height exceeds or falls below their spatial period [12]. In addition to LSFL and HSFL, supra-wavelength LIPSS have also been reported. These structures often emerge at high cumulated fluences and exhibit diverse morphologies, including coral-like [13], spikes [14], and grooves [15].

When applications require surface structures with well-controlled regularity and dimensions, such as in diffraction gratings, laser parameters must be carefully optimized to achieve the desired lateral and vertical features with sufficient uniformity. However, achieving such fine control remains challenging, as the resulting topography depends not only on the inherent regularity of the LIPSS generated within each laser focal spot but also on the chosen scanning strategy, which introduces interactions between the processing spot and the previously structured area [16]. Furthermore, despite extensive research, there is still no standardized method in the scientific community for quantifying the feature sizes and regularity of LIPSS. The most common characterization approach involves analyzing scanning electron microscopy (SEM), confocal microscopy, or atomic force microscopy (AFM) images to extract the average values and standard deviations of spatial periods and structure heights. An improvement to this procedure can be achieved by applying Fourier transform techniques to the topography images, which reveal the spatial frequency content of the LIPSS textures. Characteristic features, such as peaks or “double sickle”, can then be quantified in terms of their shape, intensity, and spectral extent, providing a spectral fingerprint of the surface morphology [17]. However, these microscopy-based techniques are typically performed ex situ and often require time-consuming sample and data acquisition, limiting their utility for real-time process monitoring.

As an alternative, scatterometry-based methods have emerged as a reliable, fast and non-destructive techniques suitable for real-time monitoring of periodic micro and nanostructures [18,19,20,21]. These optical monitoring approaches utilize the diffraction pattern, i.e., the Fourier space, produced by periodic surfaces to reconstruct key geometrical features. Scatterometry has already been successfully applied to estimate the structure depth and average spatial period of LSFLs on stainless steel under varying laser parameters, such as cumulated fluence and angle of incidence [22,23]. However, those studies did not attempt to quantify other geometrical parameters such as the uniformity or distribution of spatial periods, and were able to estimate the structure depth over a reduced range (<150 nm). Similarly, other studies have investigated the time-resolved formation of LIPSS by measuring and analyzing scattered light; however, they did not establish a methodology for quantifying spatial periods, structure heights, or pattern regularity [24,25,26,27,28,29].

In previous studies, multiscale anisotropy metrics have proven effective for analyzing directional surface textures. For example, curvature tensor analysis combined with frequency-band filtering has been used to reveal orientation patterns across multiple scales [30]. Similarly, spectral analysis techniques based on 2D power spectral density (PSD) have been proposed to evaluate directional bias, a method conceptually similar to the Fast Fourier Transform (FFT)-based isotropy analysis implemented in commercial surface analysis software [31].

In this study, a scatterometry-based method is employed to comprehensively characterize LIPSS fabricated on stainless steel substrates using picosecond laser pulses and a polygon scanner for high-speed processing. A custom-developed image processing algorithm is used to determine the type of LIPSS, structure height, spatial period, periods range, and pattern regularity, based on analysis of the scattering images and comparison with surface topographies obtained through AFM. Additionally, isotropy evaluation is utilized to quantify the directional distribution of surface features, complementing diffraction-based regularity assessments and providing an additional metric for characterizing the degree of order and orientation in laser-textured surfaces.

## 2. Materials and Methods

### 2.1. Stainless Steel Substrates

The structuring experiments were performed on stainless steel plates (X5CrNi18-10, SG Designbleche GmbH, Erkelenz, Germany) with a thickness of 0.8 mm and lateral dimensions of 80 mm × 60 mm. This austenitic chromium–nickel steel is widely used in industries, such as automotive, food processing, construction, and petrochemistry, due to its excellent corrosion resistance and good machinability [32,33]. Prior to laser structuring, the surface was electropolished to achieve a mirror finish, resulting in a surface roughness *S_q_* of 0.02 µm.

### 2.2. LIPSS Structuring

**Figure 1:**
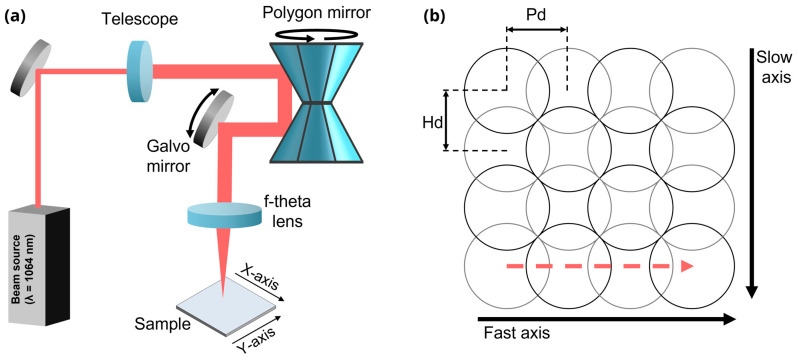
(**a**) Schematic representation of the polygon scanner setup. (**b**) Scanning strategy illustrating the difference between the fast and slow axis. The red dashed line shows the displacement of the laser spot across the fast axis, every two consecutives spots in a row are separated by a pulse-to-pulse distance indicated as *Pd*. Meanwhile every row is separated from the following one by a hatching distance indicated as *Hd*.

The laser system used for surface structuring consists of a picosecond laser source (PX600-1-GF, Edgewave, Würselen, Germany), emitting ultrashort pulses with a central wavelength of *λ* = 1064 nm and a pulse duration of *τ_P_* = 12 ps. The source supports a variable repetition rates *f_rep_* ranging from 1 kHz to 50 MHz with a maximum pulse energy of 30 μJ. A schematic of the experimental setup is illustrated in Figure 1a. The laser beam is first guided via several mirrors to a beam expander. Subsequently, the beam is directed into a polygon scanner (PMS600, MOEWE Optical Solutions GmbH, Mittweida, Germany) where it is directed along the fast axis (*y*-axis) by a rotating double-polygon mirror and along the slow axis (*x*-axis) by a galvanometer mirror. This configuration enables the system to achieve exceptionally high scanning speeds [34,35]. The beam is finally focused onto the sample using an f-theta lens with a focal length of *f* = 420 mm, resulting in a spot diameter of *d_spot_* = 40 μm. The maximum scan field has a total area of *A_scan_* = 300 × 300 mm^2^, and the maximum scan velocity that the system can achieve is *v_scan,max_* = 1000 m/s.

The processing strategy involves scanning the laser beam along the fast axis with a controlled overlap between adjacent pulses, as illustrated in Figure 1b. The pulse repetition rate was fixed at 3.28 MHz, and the scan speed along the fast axis (driven by the polygon scanner) was set to 98.4 m/s. Considering both parameters, a pulse-to-pulse distance of 30 µm is obtained, corresponding to a 25% pulse overlap in the fast-scanning direction. After completing one scan line, the beam is shifted along the slow axis (perpendicular direction, see Figure 1b) by a hatch distance of 24 µm yielding a 40% overlap in this direction. Across all experiments, the pulse energy was maintained at 18.6 μJ, while the number of scans (N) was varied from 1 to 500. The combination of both overlaps as well as the number of scans determines the cumulated fluence delivered to each sample, resulting in an estimated value of 0.82 J/cm^2^ per scan, following the methodology reported in [36].

### 2.3. Topography Characterization

The morphology of the structured surfaces was qualitatively analyzed using scanning electron microscopy (Quattro ESEM, Thermo Fischer Scientific, Hennigsdorf, Germany) operated at an acceleration voltage of 20 kV. Surface topography measurements were performed using AFM (CoreAFM, Nanosurf, Liestal, Switzerland) in tapping mode. A silicon cantilever (Tap190Al-G, Budget Sensors, Sofia, Bulgaria) with a spring constant of 48 N/m and a resonance frequency of 190 kHz, as specified by the supplier, was used for all measurements.

### 2.4. Monitoring System

**Figure 2:**
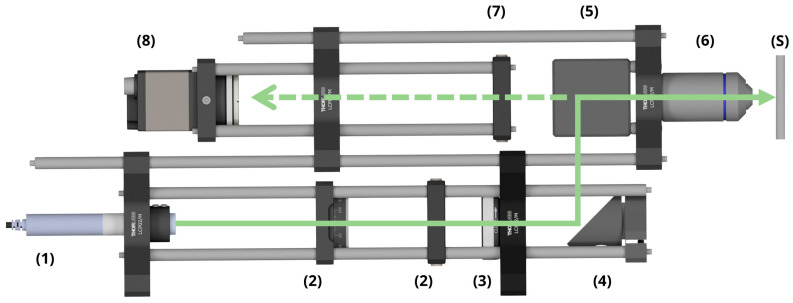
CAD drawing of the employed scatterometry setup, including all hardware components (1—laser source, 2—linear polarizer, 3—iris aperture, 4—mirror, 5—beam-splitter, 6—microscope objective, 7—lens, 8—CCD sensor, S—sample). The solid line arrow indicates the propagation of the probing beam to the sample, while the dashed line indicates the scattered light.

The monitoring system used in this work operates based on the collection and analysis of light scattered from the microstructured surface, following a design already described in a previous study [37]. A CAD rendering of the main components is shown in Figure 2. The setup uses a low-power laser diode (CPS532, Thorlabs, Bergkirchen, Germany) with a central wavelength at 532 nm (1) as the illumination source. Two linear polarizers (2) enable the adjustment of the intensity of the light reaching the camera, allowing optimal contrast during image acquisition. An iris aperture (3) is placed along the optical path to control the illuminated area on the sample. A 45° mirror (4) directs the beam to a beam-splitter (5), which transmits a portion of the light to a 50× microscope objective (6). This objective focuses the beam on the surface (S) and simultaneously collects and magnify the scattered diffraction pattern. A lens (7) focuses the diffracted light onto a CCD sensor (8). The illuminated spot on the sample surface has a diameter of 1 mm, allowing the system to capture diffraction signals from tens of thousands of LIPSS simultaneously.

Thus, the scatterometry monitoring relies on analyzing the light diffracted by the periodic surface under study. When the probe beam reaches the surface, the quasi-periodic nature of LIPSS acts as a diffraction grating, generating discrete diffraction orders (DOs) in the CCD plane [38]. The setup previously described delivers a perpendicular beam to the sample while collecting the reflected DOs described by the following grating equation:

sin(*θ_m_*) = *mλ*/*Λ* (1)

where Λ is the LIPSS period, *λ* the laser probe wavelength, m the diffraction order and *θ_m_* is the diffraction angle of the corresponding mth-order relative to the surface normal.

Since LSFL are inherently quasi-periodic, their lateral feature sizes can be described in terms of an average spatial period and an associate range. To enable this analysis, the system was calibrated so that the distance, in pixels, between the zero-order diffraction spot and a given position in the image corresponds directly to a real-space spatial period. This analysis involves the conversion of spatial frequencies to periods, taking into account the effects of the imaging optics and potential aberrations, resulting in the following equation:

*Λ* = (175.46 ± 0.64) [µm px]/Δ*x* [px] (2)

with Δ*x* being the distance in pixels of a given point to the zero DO. More details of the calibration procedure are provided in the Supplementary Information (Figure S1).

The resulting diffraction patterns exhibit characteristic intensity distributions, shapes, and positions of the diffraction orders that are unique to each specific surface topography [39]. To analyze the recorded images and estimate topography parameters from them, a custom algorithm was developed in Python 3.13.5. The algorithm extracts relevant parameters by generating intensity profiles through pixel integration within defined region of interest. These profiles are processed using modules and functions implemented on the SciPy library. Peaks and peak-to-peak distances are then identified using predefined thresholds, enabling quantitative characterization of the diffraction pattern and their correlation with the underlying surface morphology.

## 3. Results and Discussion

### 3.1. Evolution of LIPSS Topography

**Figure 3:**
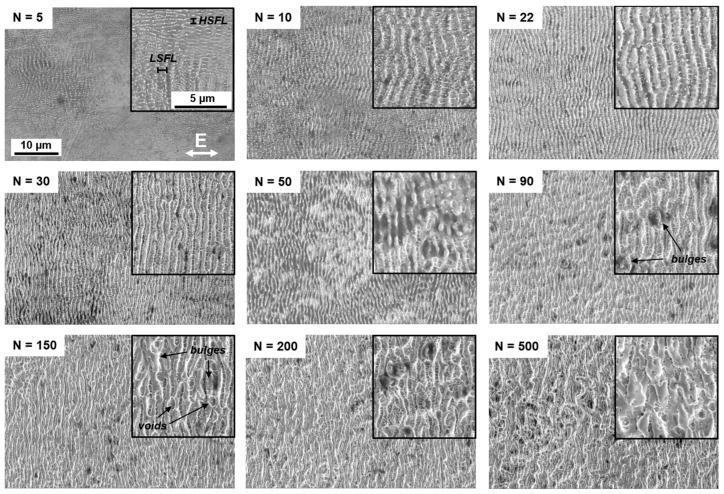
Selected SEM images illustrating the evolution on the development of LIPSS with increasing number of scans (N), having a cumulated fluence of 0.82 J/cm^2^ per each scan number.

Figure 3 presents a series of selected SEM images acquired after an increasing number of scans (see labels), aimed at investigating the evolution of LIPSS as a function of the accumulated fluence. LIPSS were not observed in samples processed with fewer than five scans. From five scans onward, the first signs of LIPSS growth become evident. At this stage, LSFL (type I) are observed with an estimated spatial period of 1130 nm, them being oriented perpendicular to the laser polarization (indicated by the double arrow in Figure 3). In addition, smaller features with a periodicity of approximately 415 nm, likely corresponding to shallow HSFL, are also visible on the same surfaces. Between approximately 10 and 50 scans, LSFL become dominant over the whole surface, while the shallow HSFL appear non-uniformly, decorating the valleys of the ripples.

As the number of scans increases from 10 to approximately 30, the LSFL appear more regular and aligned, which may be attributed to cumulative effects such as surface smoothing or enhanced feedback mechanisms during repeated irradiation. Additionally, spherical nanoparticles, likely originating from redeposited material ejected during ablation, are observed on top of the ripples. At approximately 50 scans, the surface morphology becomes less homogeneous. Although the LSFL features in coexistence with HSFL and nanoparticles are still present, new textures begin to emerge. In certain regions, the ripples appear to melt and merge, leading to re-solidified LIPSS with smoother sidewalls and a noticeable reduction in both HSFL and nanoparticles density, likely due to partial melting and material flow (see Figure 3 from *N* = 50). At approximately 90 scans, surface uniformity deteriorates further. Bulges begin to form at random positions, probably caused by the coalescence of multiple molten ripples. These new structures have a characteristic size of approximately 983 nm, which is larger than that of the initial LSFL (see indication in Figure 3 for *N* = 90, 150). When the number of scans exceeded 150, the surface texture was significantly distorted. Bulges and voids dominate the morphology, and LSFL become difficult to discern. Finally, at 500 scans, the laser-textured surface consists primarily of collapsed and molten features, with high density of bulges and voids, indicating substantial degradation of the periodic texture.

The laser-treated surfaces were also measured by AFM to acquire high resolution topography images (a selection of the AFM images is given in Figure S2, at the Supplementary Material section). Due to the quasi-periodic nature of the LIPSS, it is not possible to estimate a single, deterministic period for each texture. Therefore, in this work the 2D-FFT spectrum of the topography images was analyzed to determine the dominant frequency of the first orders and their width. Next, these values were reconverted to the real space in order to estimate the average spatial period and its range, respectively.

**Figure 4:**
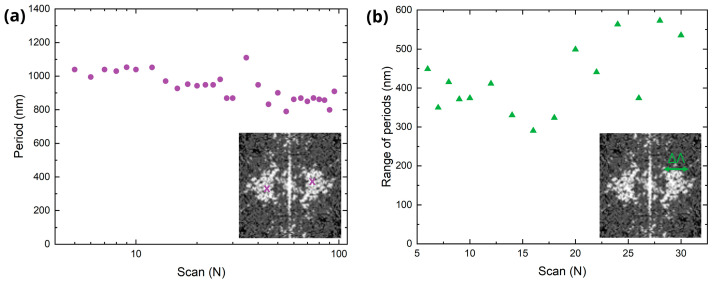
Estimation on LIPSS periods based on FFT analysis of the AFM topographies. (**a**) Average period extracted from the FFT of the topography data. (**b**) Difference between the maximum and minimum period measured in the FFT spectrum.

Figure 4a shows the calculated average period extracted from the 2D-FFT of AFM images using a Python script, while Figure 4b illustrates the range of the periods, extracted from the FFT generated with the freeware Gwyddion, version 2.66. Exemplary FFT spectra are shown as inset. The plots show that as the number of scans increases, the spatial period slightly decreases from approximately 1000 nm to 800 nm, whereas the period range tends to increase from ~400 nm up to a maximum of 580 nm. This observation might be ascribed to the melting of LSFL and appearance of the multiple sub-structures (see Figure 3, e.g., for *N* = 30, 50) that distorts the LSFL dominance and increases disorder of the textured surface.

**Figure 5:**
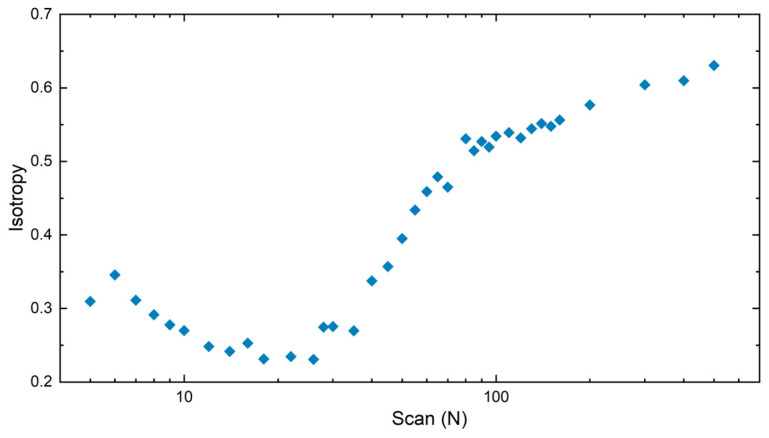
Isotropy evolution of LIPSS with number *N* of scans. A low isotropy suggests stronger directionality of the structures, while higher values indicate a more random distribution of the produced features by the laser treatment.

To estimate the regularity of the fabricated LIPSS, the isotropy of the textures was quantitatively evaluated by calculating the ISO 25175 Str parameter [40,41]. The algorithm computes the angular distribution of the PSD and quantifies the degree of isotropy by analyzing the spread and intensity of the dominant spatial frequencies. In this context, high isotropy values correspond to random or multi-directional surface features, while low isotropies indicate strong directional order, as expected for well-defined, regular, and straight LSFL. Figure 5 shows the evolution of isotropy as a function of the number of scans. Initially, the isotropy decreases from its initial value of ~0.35 (for *N* = 6) as the LSFL begin to form, indicating that the emerging structures acquire a preferential orientation. This suggests that, during the early stages of formation, the surface features lack a well-defined pattern or direction. As the process continues, the structures progressively organize in a relatively regular and straight pattern (see, e.g., Figure 3 for *N* = 22) reaching an isotropy value of ~0.23. Beyond approximately 30 scans, the isotropy increased markedly, which can be attributed to the development of grooves, bulges, and voids that disrupt the directional order of the LIPSS features.

**Figure 6:**
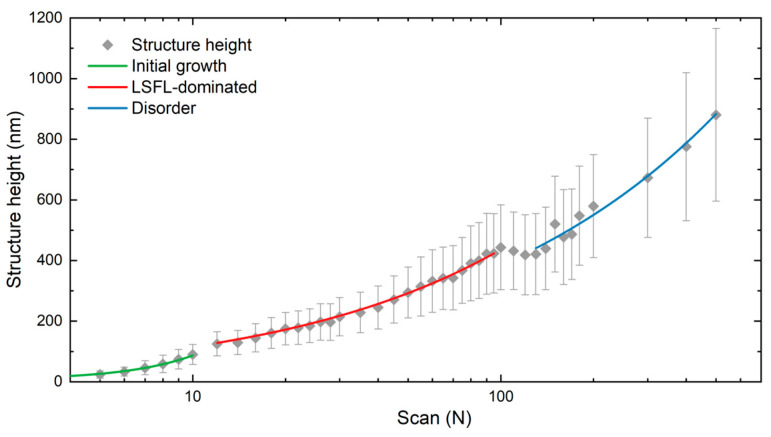
Height evolution of LIPSS using a cumulated fluence of 0.82 J/cm^2^ per each scan number. Solid lines are guides to the eye in to indicate the different identified LIPSS formation regimes (see text for details).

The average height of the structures determined by AFM is shown in Figure 6 in a logarithmic scale plot (*x*-axis) to represent the number of scans. Based on the SEM analysis described above, three different LIPSS growth regimes can be identified. The first regime, corresponding to a low number of scans (up to 10), represents the initial stage of LIPSS formation (green line in Figure 6), where the LSFL-I and shallow HSFL begin to emerge and progressively cover the laser-treated surface. The measured structure depth increases with the number of scans up to approximately 100 nm.

Between 10 and 50 scans, the LSFL homogeneously cover the treated surfaces (see Figure 3). Within this range, both the height of the LSFL and its standard deviation continue to increase, suggesting a decrease in the LIPSS uniformity. According to Figure 3, for *N* > 30 the uniformity of the LSFL degrades as new sub-structures start to emerge. However, the LIPSS height, and corresponding standard deviation, further increase until *N* = 95, reaching a height of approximately 420 nm, marking the end of the LSFL-dominated regime (red line in Figure 6). Here, a transition in the height development is observed between 95 and 130 scans, marked by a drop and subsequent plateau in structure height. This behavior is attributed to melting and merging of the ripples, which disrupts the uniformity of the LSFL coverage.

For *N* > 130 (blue line in Figure 6), the surface consists of molten LSFL, bulges, and voids (as shown in Figure 3). In this region, the structure height and its standard deviation increases rapidly reaching a maximum height of 880 ± 290 nm at *N* = 500. This regime stands out not only by a pronounced increase in height, but also by the growing disorder due to the deterioration of LSFL coverage. This regime is also characterized by a steady rise in the texture isotropy as shown in Figure 5.

### 3.2. LIPSS Characterization via Scatterometry

An optical monitoring system based on scatterometry was used in an off-line configuration to collect the diffraction patterns obtained by illuminating the laser-treated samples with the green probe laser after processing. From the characteristic patterns formed for each laser-treated surface and the corresponding intensities, different texture parameters, such as shape, spatial period, period range, structure height, and regularity, can be estimated.

For each structured area, five images were acquired at different positions to ensure statistical relevance. The exposure time of the CCD camera and polarizer orientation were kept constant throughout all measurements to avoid saturation of the zero-order diffraction signal. The exposure was adjusted based on surfaces processed with 10 scans, as this corresponds to the point at which LSFL uniformly cover the surface and become the dominant morphological feature.

**Figure 7:**
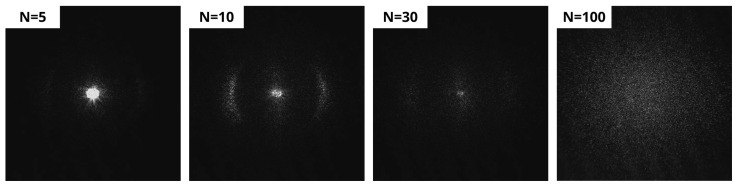
Exemplary CCD images with increasing number of scans illustrate the evolution on the studied signal. During the initial stages the signal consists of a bright central spot, then the lateral lobes start to develop and finally the signal starts to lose intensity until a diffuse cloud is formed.

Figure 7 shows representative CCD images of samples scanned 5, 10, 30, and 100 times. For the steel surface with *N* = 5, the image shows a single bright spot at the center, corresponding to the zero-order diffraction, without any significant features that could indicate the presence of LIPSS. At *N* = 7, the diffraction pattern displays its characteristic double-sickle shape, which corresponds to the first positive and negative diffraction orders (+1 and −1) in Fourier space, typically associated with LSFL [42]. With an increased number of scans (e.g., *N* = 10), the first-order diffraction features become more pronounced, indicating the enhanced surface coverage and improved regularity of LSFL structures. No features corresponding to diffraction from HSFL could be detected by the CCD camera, because their spatial period (~400 nm according to the SEM images in Figure 3) was shorter than the illumination wavelength (532 nm).

However, at approximately 30 scans, the intensity of the double-sickle shape pattern starts to diminish and eventually becomes barely visible. Several factors could explain this behavior, the most prominent being the reduction in diffraction efficiency of reflective diffraction gratings as the LIPSS height increases. In particular, it is well-known that diffraction efficiency is highly sensitive to the structure height, particularly in the visible spectrum, where the maximum efficiency typically occurs for heights between 100 and 200 nm [43]. As shown in Figure 6, the LIPSS height increases further with the number of scans, reaching approximately 330–400 nm at *N* = 60, which likely results in significantly reduced diffraction efficiency. Additionally, changes in surface chemistry, such as local oxidation caused by increased thermal input, may also contribute to the reduction in the measured signal. The formation of oxide layers can act as anti-reflection coatings, reducing the intensity of the reflected light and weakening the detectable diffraction orders.

At higher scan values, such as *N* = 100, the central diffraction spot (Figure 7) appears broader in both horizontal and vertical directions, forming a cloud pattern. The broadening is attributed to the formation of bulges and randomly distributed molten material, which scatter the light diffusely rather than directing it into distinct diffraction orders, thereby diminishing the structured pattern in the Fourier space.

**Figure 8:**
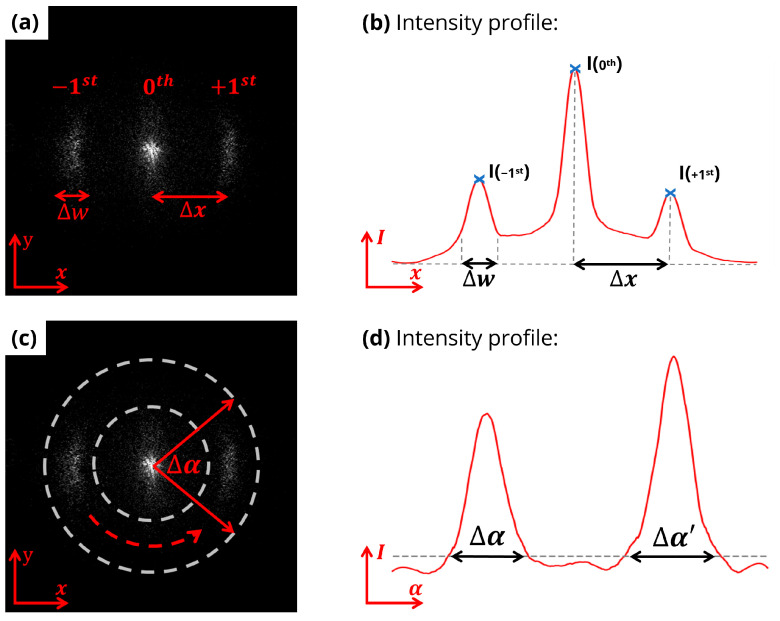
Profile extraction from a single image. (**a**,**b**) Longitudinal profile highlighting DO peaks, Δ*x* indicates the distance to the zero DO and Δ*w* the width of the DO, later used to estimate the main period and its range. (**c**,**d**) Circular profile revealing the angular spreading of the DO, Δ*α* represents the opening angle measured from the profile.

In order to extract information about the texture features from the recorded images, intensity profiles were generated by compressing the image, reducing the diffraction patterns to a one-dimensional signal. Figure 8 illustrates the extraction of two distinct types of profiles derived from a single image. In the first example (Figure 8a,b), the profile is generated by adding up the pixel intensity of the image along the vertical axis. This process enables the acquisition of profiles with well-defined peaks (Figure 8b) corresponding to each DO, allowing for the straightforward extraction of peak intensity, their distance to the zero DO and their width. To evaluate the angular intensity distribution of the first DOs, two concentric rings (white dashed lines in Figure 8c) were defined and the intensity enclosed between them was added up along the direction indicated by the dashed arrow. As a result, intensity profiles along a 360° arc were extracted, thereby enabling a direct measurement of the opening angle with a fixed threshold (Figure 8d).

To estimate the average LSFL period, the positions of the first-order peaks were extracted from the intensity profile using the custom signal processing algorithm and next converted from distances between the first and zero DO to spatial periods. The dispersion of the diffraction orders was evaluated by measuring the width of the peaks and converting the starting and ending point of the width to real space. Therefore, the maximal and minimal periods are obtained, and their difference gives the dispersion, or range, of spatial periods.

**Figure 9:**
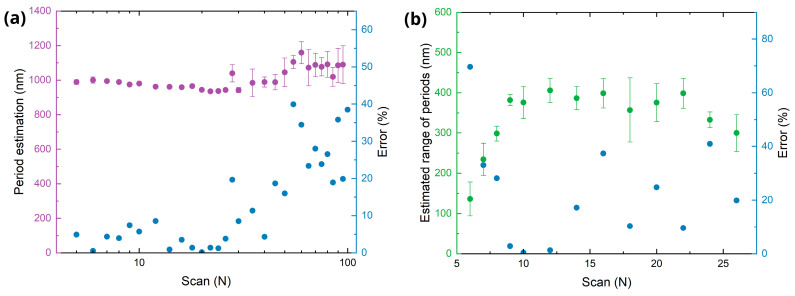
Estimation of LIPSS periods. (**a**) Average period (left axis, violet) estimated by the scatterometry setup and the relative error (right axis, blue) compared to AFM measurement (error). (**b**) Estimated range of period (left axis, green) and its error compared to the AFM measurements (right axis, blue).

Figure 9 summarizes the estimated period and range (left axis), and the magnitude of their relative error (right axis) when compared to the corresponding values obtained from the FFT of the AFM images (shown in Figure 4). The results show good agreement between scatterometric and topographic measurements up to 30 scans, with a relative error below 10%. When applying more than 40 scans, the formation of a diffuse central diffraction pattern, referred to as a cloud, along with a drop in diffraction efficiency, reduces the accuracy of the scatterometry method for estimating the spatial period. The estimation of the range is less accurate than the estimation of the period probably due to the arbitrary determination of thresholds for calculating the width of first DO in the CCD images and 2D-FFT of AFM images.

To evaluate the regularity of the LIPSS, the angular spread, or opening angle Δ*α* (Figure 8c), of the first DO in the Fourier space is calculated [44]. A smaller opening angle indicates straighter and more regular LIPSS, as the frequency components of the first DOs are concentrated in well-defined spots aligned along the horizontal axis. Contrary to this, a larger opening angle indicates wavier and ripple-like LIPSS, with a spectral content dispersed over the horizontal and vertical axes [45,46]. For a quantitative analysis, intensity profiles were generated by integrating pixel values between two concentric rings surrounding the first (+1 and −1) DOs (see Figure 8c,d), while excluding the central specular reflection, creating thus a band pass filter between 666 nm and 1200 nm. The opening angles were measured from each of these profiles based on a fixed threshold and then averaged for each image.

**Figure 10:**
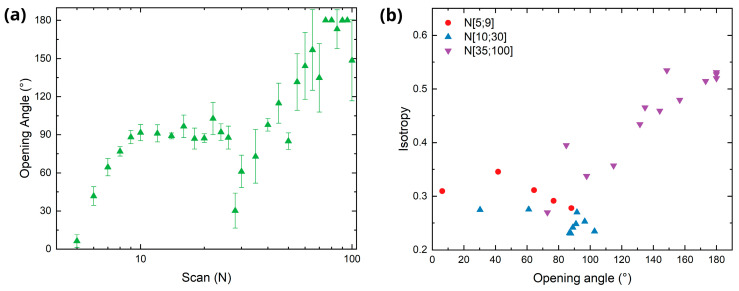
Estimation of LIPSS regularity. (**a**) Evolution of the opening angle with increasing number of scans. (**b**) Isotropy as a function of the opening angle.

Figure 10a shows the evolution of the opening angle as a function of scan number. As the LIPSS start to form, the opening angle increases up to a stable value of approximately 90° for 9 to 26 scans. After this plateau, the opening angle suddenly drops implying more regular structures, such as the texture shown in Figure 3, for *N* = 30. As *N* increases further, the opening angle rises constantly, which can be associated with the behavior observed in Figure 7 for *N* = 30 and 100, where the intensity drops and the diffuse cloud begins to form as the scan number exceeds 30. This transition correlates with the appearance of new structural features around the LSFL due to high cumulated fluence (as already explained in the SEM analysis of Figure 3), which further degrade the periodic order of the surface confirmed by the increase in the opening angle.

The isotropy values previously obtained (Figure 5) were compared with the opening angle, as shown in Figure 10b. During the initial stage of LIPSS formation (*N* < 10, red circles) and the subsequent development of LSFL (10 < *N* < 30, blue triangles), no strong correlation is observed between isotropy and opening angle, suggesting that LIPSS regularity cannot be reliably estimated from scatterometry measurements in these regimes. The only relevant information that can be obtained from such measurements is that opening angles below ~100° strongly indicate the presence of LSFL with isotropy values ranging between 0.25 and 0.35. For *N* > 30 (purple triangles), an approximately linear trend could be observed, reflecting the emergence of sub-structures and the gradual degradation of periodicity. As a result, a measured opening angle higher than 120° hints at the presence of voids, bulges, and molten ripples over the LSFL.

**Figure 11:**
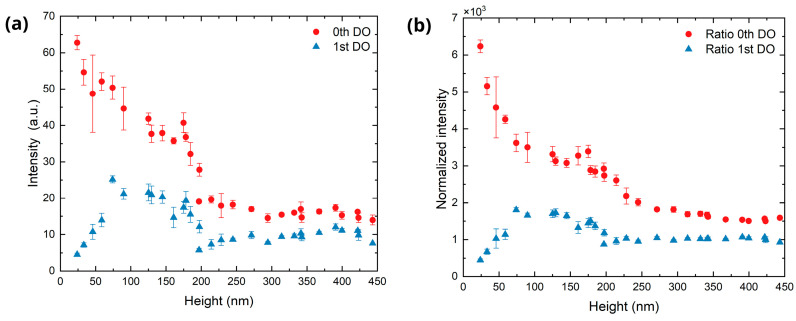
Diffraction orders peak intensity behavior as a function of the LIPSS height. (**a**) Peak intensity extracted from the profile intensity. (**b**) Ratio between the peak intensity of the DO and the average pixel intensity of the CCD image.

As reported in previous studies [47], the height of the LIPSS can be inferred from the intensities of the zero and first diffraction orders recorded in the scatterometry images. To this end, their peak intensities were extracted from the intensity profiles, and plotted as a function of the structure height, as shown in Figure 11a. In the case of first (+1 and −1) DOs, their intensities were averaged. It can be seen that the first-order intensity increased with the structure height, reaching a maximum at 74 nm and then remaining relatively constant. Then, intensity starts decrease at 175 nm, reaching a minimum at 196 nm, after which no significant variation on the intensity is seen. In contrast, the zero-order intensity decreases continuously as the structure height increases. Beyond 196 nm, the zero DO intensity follows the same behavior as the first order, remaining approximately constant.

As discussed previously, upon increasing the number of scans not only the LSFL tend to cover the treated surface, but also other sub-structures appear, such as bulges, voids, or nanoparticles. Due to this, the double-sickle pattern typical of LSFL becomes less defined (as mentioned before at *N* = 30), and a cloud forms around the central spot (see, for example, Figure 7, *N* = 100). Furthermore, this cloud observed in the diffraction pattern can be ascribed to randomly scattered light. As a consequence, the accuracy of height estimation for LIPSS fabricated for scans *N* higher than 30 (equivalent to a structure height larger than 200 nm) deteriorates when relying solely on the analysis of the zero and first diffraction order intensities, as these intensities remain constant in this scan range (see Figure 11a).

This limitation can be addressed by considering the total intensity of the image as an additional metric for the randomly scattered light within the region of interest. While the intensities of both the zero and first diffraction orders exhibit a steep decline at *N* = 30 (corresponding to a structure height of approximately 200 nm), the total integrated intensity of the image begins to increase beyond this point, reaching a new maximum at approximately *N* = 80 (see Figure S4a in the Supplementary Information). To extend the estimation capability of the structure height into the cloud formation regime, a new parameter called normalized intensity (*I_norm_*) is defined as the ratio between the intensity of the DO and the average pixel intensity of the full image, as indicated in the following equation:

(3) $$I_{norm}=I_{DO}/\sum_{i,j}^{m,n}\frac{I_{px}\left(i,j\right)}{m\;n}$$

where *I_DO_* indicates the intensity of the DO, *m* and *n* the dimension of the image in pixels and *I_px_* the pixel intensity. Figure 11b displays this ratio for the zero and first order as a function of LIPSS height. Remarkably, in the case of the zero-order a monotonous decrease is observed for heights in the range 200–400 nm, enabling the establishment of a correlation between this parameter and LIPSS height during the latest stage of development.

**Figure 12:**
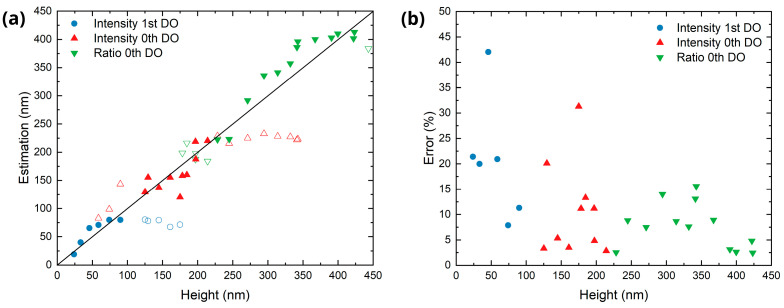
Estimation of the structure height from a set of scatterometry images. (**a**) Comparison between the estimated and measured (by AFM) structure height using three estimation methods (different colors and symbols). The hollow symbols indicate points out of the corresponding working range. (**b**) Magnitude of the relative error between the estimated and measured height.

To estimate the LIPSS height from the intensities of the zero and first DOs, three separate fitting strategies were used, each tailored to one of the identified ranges. Figure S4b–d shows the obtained fits using four CCD images for each number of scans, while Figure 12a shows the evaluation of the remaining CCD images in order to test the structure height estimation. Figure 12b shows the magnitude of the relative error of this estimation, i.e., using this specific image set, compared to the AFM measurements displayed in Figure 6. Namely, for the initial formation stage (*N* < 10), it was found that using the fitting for the intensity of the first DO minimizes the average estimation error to 20.6%.

For the range in which the LSFL are dominant on the treated surfaces (10 < *N* < 30), fitting the zero-diffraction order allows for the best estimation of the LIPSS height with an average error of 10.7%. Finally, in the cloud formation regime, i.e., *N* > 30, using the fit for the normalized intensity of the zero-order yield the lowest average error (7.7%). Consequently, the presented scatterometry approach is able to estimate LIPSS at different formation stages and in a relatively broad range of structures heights from 25 nm to 420 nm.

The methods and techniques employed for monitoring LSFL height represent a significant improvement on previous work, in which the measurable range was limited to 140 nm [23]. Although the compact design limits the period estimation resolution, compared to similar approaches [22], it enables the system to be integrated into industrial environments. Furthermore, the information provided by the opening angles offers valuable insights into the dominant type of LIPSS under inspection. The presented results demonstrate a substantial improvement on compact monitoring systems for LIPSS, which are highly valuable for ensuring quality and control in industrial environments.

## 4. Conclusions

This study demonstrated that scatterometry is a viable, non-destructive method for monitoring the topographical features of LIPSS on stainless steel surfaces processed with ultrashort pulsed lasers. By analyzing the scatterometry images, key surface parameters such as shape, height, spatial period, and pattern regularity of LIPSS at different formation stages were estimated obtaining the following results:

- Not only was the double-sickle shape characteristic of LSFL detected in the CCD images, but also the formation of a cloud overlapping, and eventually, dominating over the double sickle was observed. This allows for the qualitative estimation of the type of structures present on the surface.
- The estimated periods presented an average error of 5.1% up to 40 scans, while the range of periods had an average error of 22.7% for 6 to 26 scans. These ranges are characterized by clearly defined first DO in the CCD images.
- By analyzing the relationship between the opening angle and the isotropy index, a correlation was observed between these two parameters. This made it possible to establish a threshold for the opening angle that indicates either the predominance of regular LSFL or high levels of texture disorder due to the presence of multiple sub-structures.
- Using three fits correlating the intensities of the DO as function of structure height for different regimes, it was possible to estimate LIPSS heights in a range from 25 nm to 420 nm, with an average error of 11.4%. Furthermore, the evaluation of this approach indicates a decreasing error with increasing LIPSS heights.

The results confirm that scatterometry-based analysis correlates well with reference measurements obtained by AFM and SEM, especially during the early and intermediate stages of LIPSS formation. Future works may explore the integration of scatterometry into industrial laser processing systems and the limitations as a real-time monitoring approach for ensuring quality and consistency on micro- and nanostructured surfaces.

## Data Availability

The data presented in this study are available on request from the corresponding author.

## Supplementary Materials

The following supporting information can be downloaded at: https://www.mdpi.com/article/10.3390/s25165031/s1. Figure S1: Calibration fit for the scatterometry setup; Figure S2: AFM images of produced LIPSS at different scan numbers; Figure S3: AFM FFT images; Figure S4: Height estimation of LIPSS.
