# Supplementary material for: Scatterometry-Based Monitoring of Laser-Induced Periodic Surface Structures on Stainless Steel
Source: Sensors (Basel). 2025 Aug 13;25(16):5031. doi: 10.3390/s25165031 (PMC12390449; doi:10.3390/s25165031)
Supplement: Supplementary file 1 [file sensors-25-05031-s001.zip › sensors-3803741-supplementary.pdf]

sensors

Supplementary Information

Calibration of Scatterometry Setup

For establishing a correlation between the size of the LIPSS and the observed diffraction patterns, a calibration function was generated. For this purpose, steel samples with line-like structures were observed under the scatterometry setup. This samples were manufactured previously by using direct laser interference patterning, giving well defined periodic structures to the surfaces and a high homogeneity degree. After examination of this samples under the scatterometry setup, well defined diffraction patterns were observed, allowing to extract the distance between the first and zero DO. Figure S1 shows the relation between the distance of the first DO to the zero and the structure period on a reciprocal scale.

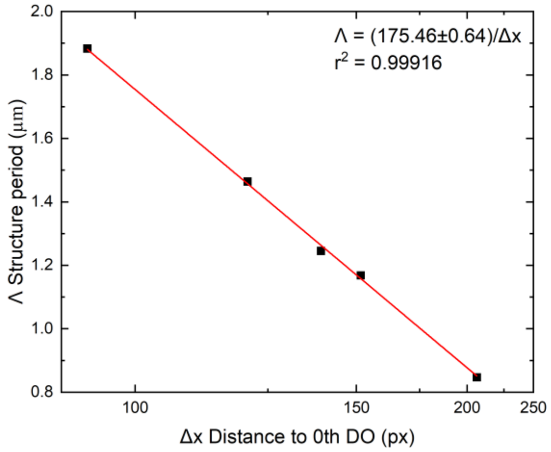

Figure S1. Calibration fit for the scatterometry setup. The parameter r^2 indicates the coefficient of determination of the fitting function.

AFM Imaging of LIPSS

To complement the SEM analysis, AFM was performed on the treated surfaces. The topographical images allow the height of the LSFL to be extracted and perform a comparative analysis between the estimations obtained by the scatterometry setup and those obtained using standard measuring devices. Figure S2 displays a selection of AFM images of LIPSS structures.

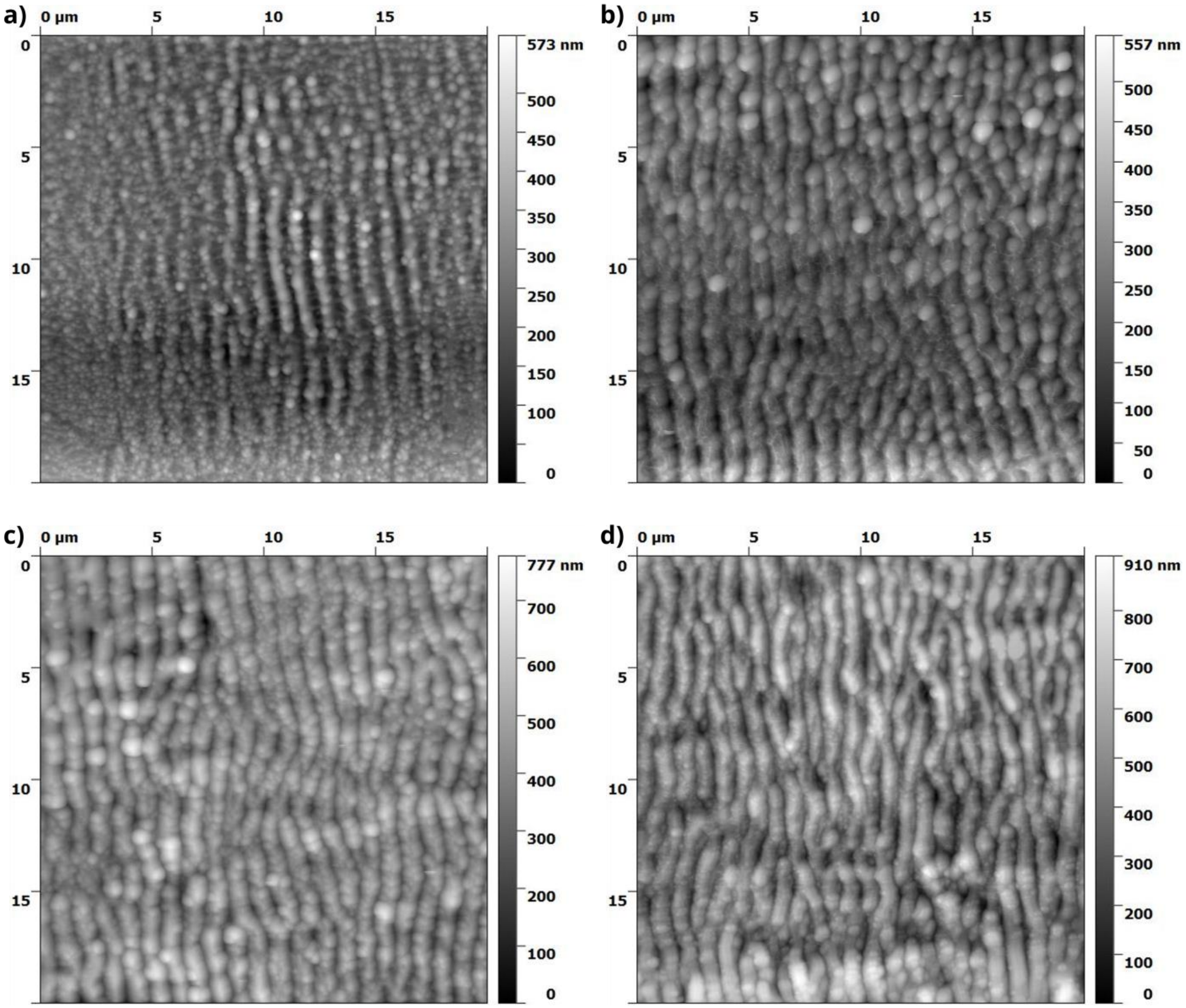

Figure S2. Selection of AFM images with: a) 6, b) 12, c) 24, d) 50 scans, where the cumulated fluence increases 0.82 J/cm^2 for each scan.

FFT Analysis of AFM Images

To obtain quantitative information of the surfaces under study and compare them with the retrieved information from the scatterometry setup, AFM images were taken and analyzed using the free software Gwyddion. Figure S3 illustrates some examples of FFT images of different scan numbers, indicating changes in the formation of the LIPSS.

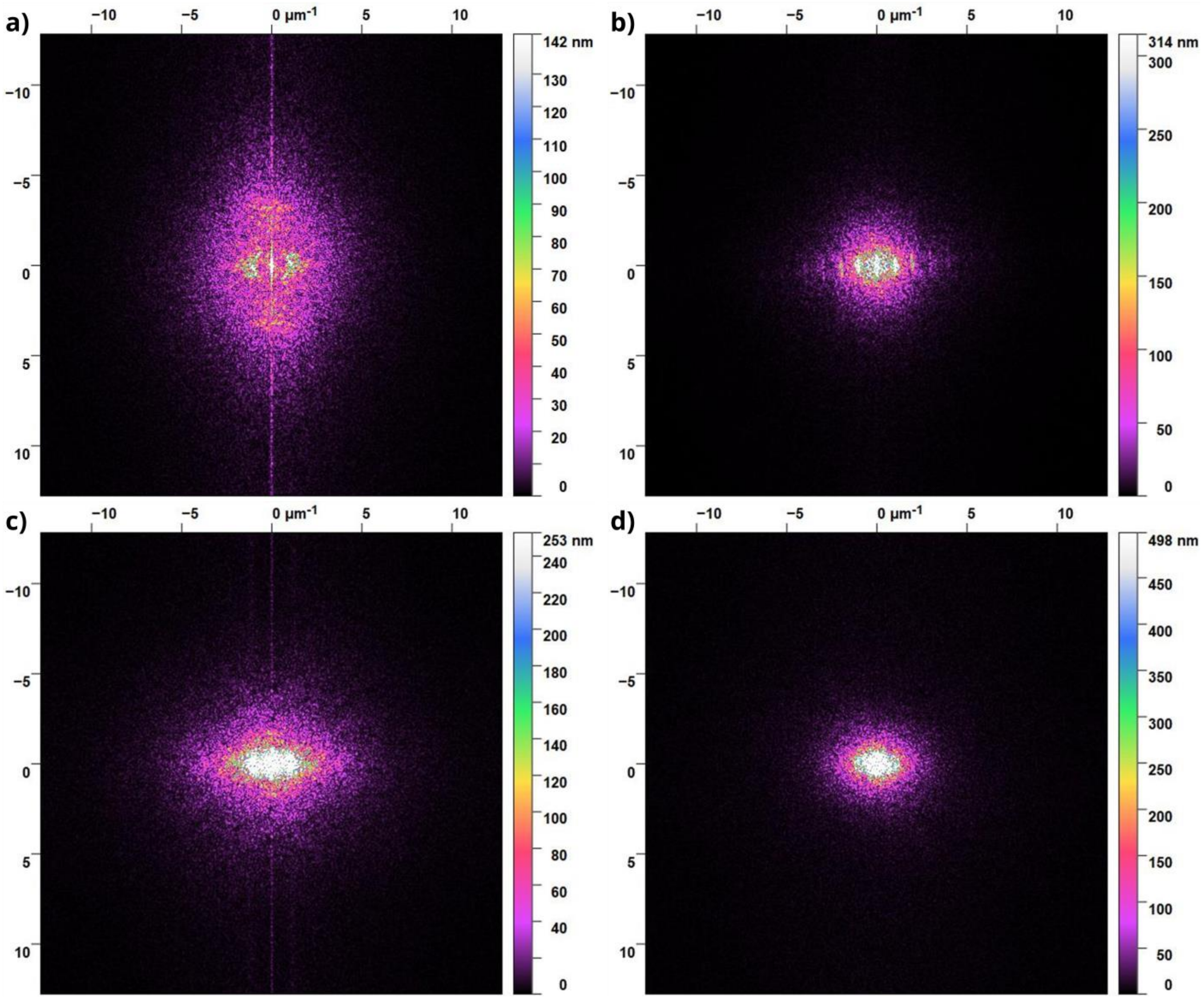

Figure S3. AFM FFT images of a) 5, b) 26, c) 50 and d) 100 scans.

Height Estimation

In order to monitor the constant height development of the LIPSS as the number of scans increase, different variables were used to stablish correlations in specific growing stages. Figure S4a shows the average pixel intensity, while Figure S4b-d show three different fits designed to monitor LIPSS height in the initial development and in the LSFL dominant regime in two different ranges.

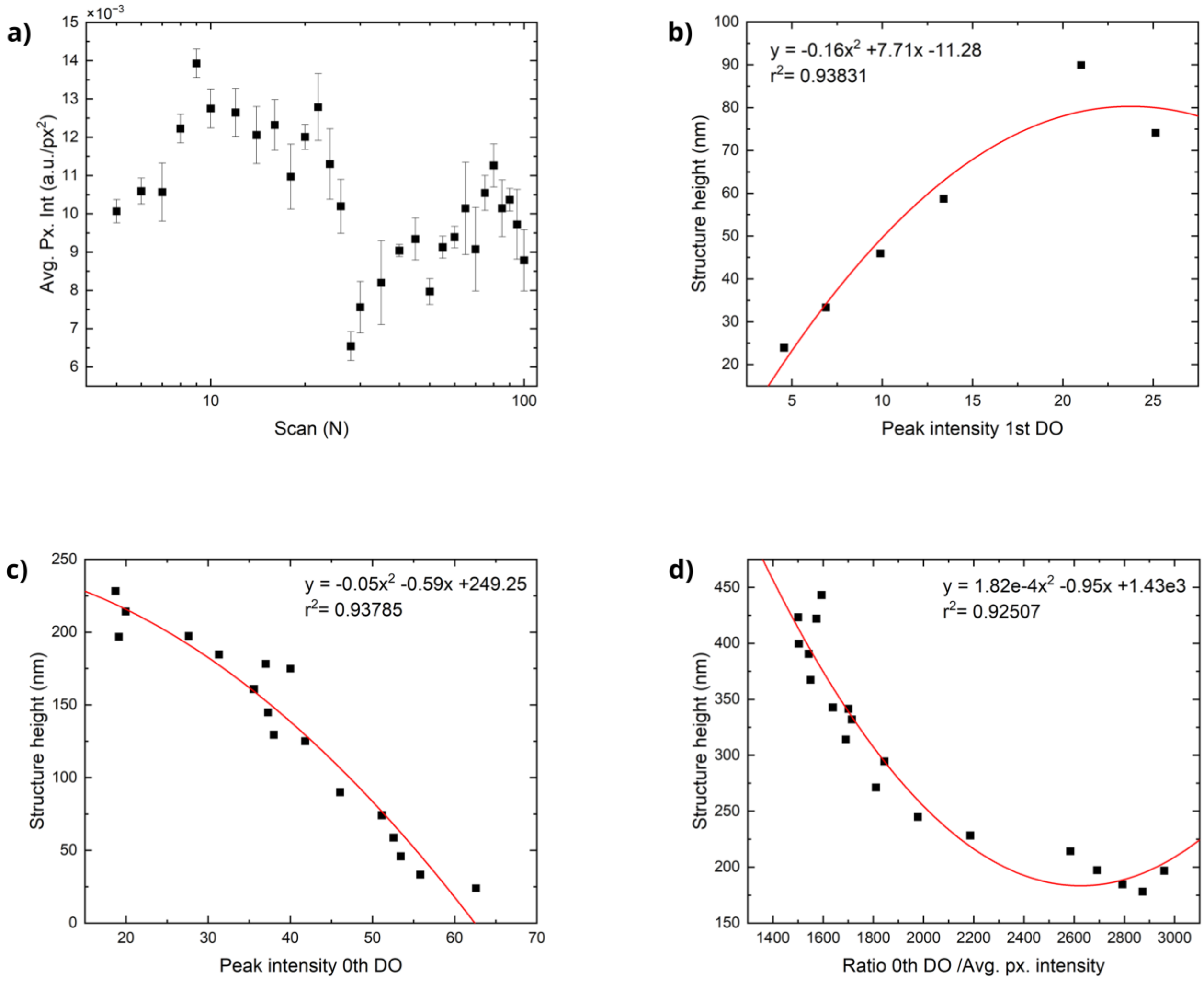

Figure S4. Height estimation of LIPSS. a) Average pixel intensity for each scan number. b-d) Fits for height monitoring employing first DO intensity, zero DO intensity and the ratio between zero DO and the average pixel intensity. The r^2 parameter of the fitting functions indicates the coefficient of determination of the functions.
